# Seasonal divergence in reproductive timing on the verge of spring: comparing hypothalamic transcriptome of two seasonally sympatric North American songbird populations

**DOI:** 10.3389/fphys.2025.1627516

**Published:** 2025-09-03

**Authors:** Devraj Singh, Adam M. Fudickar, Ellen D. Ketterson

**Affiliations:** ^1^ Biology Department, University of Kentucky, Lexington, KY, United States; ^2^ Biology Department, Indiana University, Bloomington, IN, United States; ^3^ Environmental Resilience Institute, Indiana University Bloomington, Bloomington, IN, United States

**Keywords:** seasonal reproduction, stable hydrogen isotope, migration, dark-eyed junco, hypothalamus, gnrh1

## Abstract

Every year as spring approaches and day length increases, many birds begin to reproduce, an annual expression of seasonal phenology that requires physiological preparation. In species distributed over a broad geographic range, populations that breed at higher latitudes are often migratory and delay reproduction until later in the year as compared to those breeding at lower latitudes. Dark-eyed Juncos serve as an excellent model for understanding the timing mechanisms regulating population-level variation in seasonal reproductive responses. We compared two seasonally sympatric dark-eyed junco populations in early spring. One migrates (*Junco hyemalis hyemalis*) and breeds in Alaska and Canada, while the other remains resident (*Junco hyemalis carolinensis*) and breeds in the Appalachian Mountains of Virginia USA. These populations exhibit different photoperiodic responses to the same environment with respect to activation of the HPG axis, leading to earlier gonadal recrudescence in the resident population. We caught co-wintering sympatric male migrant (n = 6) and resident (n = 7) juncos from the field in March and collected the hypothalamic tissues. We also collected blood samples to determine circulating testosterone and a wing feather to determine stable isotope ratios (δ^2^H) as estimate of breeding latitude. We found three differentially expressed genes, among which gonadotropin releasing hormone 1 (GnRH1) showed significantly higher expression in early breeding residents as compared to migrant juncos. The δ^2^H showed a positive linear correlation with testosterone levels and GnRH1 mRNA, providing strong evidence for latitudinal variation in breeding phenology. This study provides insight into the underlying neuroendocrine response giving rise to a population-level difference in the timing of reproduction observed in a seasonally sympatric (co-wintering) population of resident and migrant juncos.

## Introduction

Seasonal animals have evolved different timing strategies to match their recurring physiology of migration and reproduction to the environment (Ketterson and Greives, 2025). Annual changes in day length (photoperiod) are a primary entraining cue for endogenous clocks to synchronize with the environment. Avian populations that live in sympatry during the non-breeding state from winter to early spring may differ in whether or not they migrate. If they do, then they may breed at different locations in spring and summer, thus becoming allopatric (Fudickar et al., 2016; Ramenofsky et al., 2017; Wanamaker et al., 2020). One such species is the dark-eyed junco, which is distributed over a broad geographic range. In the junco, closely related populations winter in sympatry but differ in when they breed and whether they migrate. This pattern of sympatry in winter and allochrony in breeding makes the dark-eyed junco a powerful model to investigate the neuroendocrine basis of differences in the timing of reproduction because they pass through an overwintering period together and start to differ in their behavioral and physiological responses to similar environmental cues as spring approaches.

Use of hydrogen isotope signatures in the movement ecology of birds is a common tool in practice to predict breeding latitude (Fudickar et al., 2016; Wanamaker et al., 2020; Singh et al., 2021). Birds incorporate isotope signatures through their diet or nearby water bodies (Cormie et al., 1994; Langin et al., 2007). The local isotopic signature is incorporated into the growing feather during the post breeding molt. Because feathers are epidermal growths that are metabolically inactive, they later reflect the local isotopic signature from the geographical location where they were grown (Hobson, 1999). Stable isotopes such as hydrogen vary geographically and are used to estimate the breeding location and location of feather growth (Hobson, 2005; Powell and Hobson, 2006; Girvan et al., 2007; Maggini et al., 2016). In the case of the junco, migrant and resident populations can be distinguished during winter by the lighter hydrogen stable isotope values in migrants as compared to the heavier values found in residents.

Songbirds undergo seasonal recrudescence in their reproductive processes, primarily regulated by hypothalamic GnRH neurons (Li et al., 1994; Stevenson et al., 2009). In the temperate zone, birds initiate their gonadal development in response to increasing day lengths during early spring. However, the time of reproductive response varies depending on breeding latitude. The seasonal reproductive response is induced primarily through the interaction of increasing day length with the hypothalamic-pituitary-gonadal (HPG) axis (Li et al., 1994; Cho et al., 1998). After the winter solstice, the photoperiod starts to increase, which stimulates the HPG on reaching a population-specific threshold level of photoperiod (Wanamaker et al., 2020; Singh et al., 2021). The annual change in day length varies with latitude, such that birds breeding at higher latitudes often require longer days to initiate gonadal recrudescence than birds breeding at lower latitudes (Dawson, 2013; Fudickar et al., 2016; Singh et al., 2021). The birds breeding at lower latitudes recrudesce gonads earlier, stay in the stimulatory phase for a longer period, and terminate breeding later than high latitude birds that have a short breeding period (Singh et al., 2021).

In studies done on populations that overwinter in sympatry and breed at different locations, the higher latitude breeding birds have to accommodate an additional life-history event of spring migration that delays their reproduction (Greives et al., 2016; Fudickar et al., 2016; Ramenofsky et al., 2017). This process of spring migratory preparedness involves seasonal pre-migratory fattening, increase in food intake, and an increase in metabolic activity in flight muscles and liver in phenology and behavior (Sharma et al., 2022; Fudickar et al., 2016; Singh et al., 2018). There is also a striking shift in the locomotor activity of migratory birds, switching from only day activity to nighttime activity (Gwinner and Czeschlik, 1978; Berthold, 1996; Singh et al., 2015; Singh and Kumar, 2017; Singh et al., 2018). These changes in behavior and physiology associated with seasonal induction of reproduction and migration are potentially an outcome of some key RNA molecules changing their expression in the hypothalamus in response to changes in spring day length and other timing cues (Singh et al., 2015; Mishra et al., 2016; Johnston et al., 2016). The hypothalamus along with the pituitary, is a key neural center that synthesizes and releases neuropeptides and hormones critical to the regulation of seasonal reproductive and migratory processes. A key step in the induction of the gonad recrudescence is initiated by release of gonadotropin releasing hormone 1 (GnRH1) from the hypothalamus followed by its binding to the pituitary gonadotroph to stimulate release of luteinizing hormone (LH) and follicle-stimulating hormone (FSH) (Li et al., 1994). Investigating molecules involved in gonad growth, production of sex steroids, energy homeostasis and metabolism may help advance our understanding of differences in the reproductive timing of songbirds breeding at different latitudes.

The Dark-eyed junco provides a powerful model system for investigating the neuroendocrine mechanisms that underlie variation in reproductive and migratory timing. In early spring, populations of juncos that differ in whether they migrate are found living together in the same environment (Greives et al., 2016; Wanamaker et al., 2020). As spring progresses, the locally breeding population undergoes gonadal recrudescence, while the migratory population delays recrudescence to prepare to migrate. In a common garden study on resident and migrant juncos held in captivity, neuroendocrine tissues collected during the reproductive divergence state revealed differential patterns of gene and protein expression between residents and migrants (Fudickar et al., 2016; Bauer et al., 2018; Singh et al., 2020). However, a comprehensive account of the transcriptome associated with divergent state physiology in neuroendocrine tissue is needed to better understand the underlying mechanisms regulating early spring-induced phenologies.

The objective of this study was to discover key transcripts in the hypothalamus tissue of free-living juncos on the verge of divergence in early spring. Prior studies (Fudicker et al., 2016) conducted on captives provided insight into the mechanisms through which population-level seasonal divergence is set in a controlled indoor condition, including differentially expressed genes in muscle, blood, and hypothalamus. The present study asks how the populations differ in their hypothalamic gene expression when sampled directly from their natural environment during the time of seasonal divergence.

The following hypothesis to understand how neuroendocrine response will determine the timing of reproduction in junco populations on the verge of divergence: 1) We predicted to find differences in the plasma testosterone levels as a physiological measure of gonadal recrudescence. 2) We predicted to see the differences in the key hypothalamic transcripts associated with the latitude of breeding. 3) We predicted to see differential expression of hypothalamic transcripts associated with migratory preparedness. To test these hypotheses, we sampled sympatric resident/migrant birds that breed at different latitudes and flock together during early spring with a pre-notion of individual variation in response to constantly changing natural day length and climatic conditions.

## Materials and methods

### The study system

**FIGURE 1:**
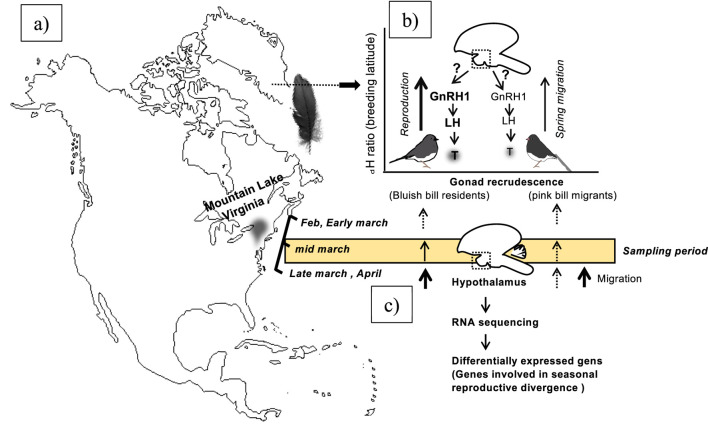
Schematic showing the wintering ground location of sympatric resident (*J. h. carolinesis*; bluish bill) and migrant (*J. h. hyemalis*; pink bill based on IUCN, 2019) dark-eyed juncos on the North America map **(a,b)**. The right upper schematic figure shows early spring difference in gonad development between early breeding resident juncos compared to migrants when captured on their wintering ground **(b)**. The lower right schematic figure shows the flow-chart outline steps of RNA sequencing, differentially expressed genes (DEGs) analysis **(c)**.

The dark-eyed junco subspecies live in sympatry and flock together at Mountain Lake Biological Station (MLBS), Virginia, from early fall to spring and start to diverge as soon as the photoperiod starts increasing (Figures 1a,b). In the wintering ground, the resident and migrant juncos are identified by their beak color. The migrants are pink-billed, and residents have blue-billed resident birds that breed at MLBS (Greives et al., 2016; Fudickar et al., 2016). The migrant juncos start to prepare for departure to the North as day length increases. Whereas resident juncos start showing gonadal recrudescence (Figure 1).

#### Body mass and Fat score

After taking the bird out of the mist net, we visually estimated subcutaneous fat score (0–5) deposition in the furcular and abdominal regions, as follows: no fat present (0), trace amounts of fat in the furcular region (1), trace amounts of fat in the abdominal region (2), half full in both furcular and abdomen area (3), full in both the areas (4), bulging in both the areas (5). Body mass was measured using a Pesola Spring scale (0–50 g), tared to zero with a small piece of sock used to carry the bird for body mass measurement.

#### Capture and tissue collection

We captured six migrants and seven resident male dark-eyed juncos using mist nets from state road 714, Giles County Virginia at Mountain Lake Biological Station (MLBS; 37.37 0N, 80.52 0W). Migrants (J. h hyemalis) and residents (J. h carolinensis) were identified using bill coloration, plumage, and wing cord (Ketterson and Nolan, 1976). Birds were caught from 13 to 20 March 2017 during spring initiation at MLBS (11.72 ± 0.1 h of average day length and −1 to −4 °C average daily temperature). Juncos were euthanized using isoflurane within 5 min from the time they were caught in the mist net. Before euthanizing, 100 μL of blood was collected by puncturing the wing vein for baseline testosterone hormone measurement. After the capture of the birds, the brain and testes were immediately dissected within 5 min, and tissues were flash-frozen on dry ice and stored at −80 °C until further processing of tissues. Scientific collecting permits were issued by the Virginia Department of Game and Inland Fisheries (permit # 052971). All methods were approved under a protocol (# 15-026-17) by the Indiana University Institutional Animal Care and Use Committee.

The hypothalamic area was dissected from the whole brain under the cryostat (Leica Biosystems CM1850, Buffalo Grove, IL, United States) maintained at −20 °C using a modified protocol adapted from Ashley et al. (2014). The frozen brain was sliced on the rostral caudal position until we saw the tractus septomesencephalicus (TrSM) as a landmark for the beginning of the hypothalamus. We sliced 30 μm thick sections and punched directly above the optic chiasma using a sterile 3 mm-diameter biopsy punch (Thermo Fisher Scientific, Integra™ 3332; Cat no. 12-460-406). All the tissue punches starting from TrSM to the end of the infundibular nuclear complex (INc) as a posterior landmark for the hypothalamus were collected in a sterile 1.5 mL eppendorf tube kept in cryostat throughout brain punching (Singh and Kumar, 2017; Mishra et al., 2016; Singh et al., 2023).

### Stable hydrogen isotope analyses

The distal most secondary feather from each individual was collected and stored in the coin envelopes and transported to the Indiana University, Bloomington. All feathers were cleaned using a chloroform: methanol in a 2:1 ratio to remove any external contaminants. A small fraction from the tip of the feather was cut, weighed to approximately 0.5 mg, placed in a 3 × 5 mm silver capsule, and shipped to a laboratory located in the US Geological Survey in Denver, CO, to analyze the hydrogen isotope (δ^2^H) values. Hydrogen isotope ratios were measured using established methods of mass spectrometry (Wunder et al., 2012; Fudickar et al., 2016; Singh et al., 2021; Wanamaker et al., 2020). The non-exchangeable δ^2^H values were reported in parts per mil notation (‰) with respect to VSMOW (Vienna Standard Mean Oceanic Water) using Caribou (−157‰) and Kudu (−35.3‰) standards. We used δ^2^H values to estimate the latitude and photoperiod where each bird spent the breeding, molting season and incorporated local δ^2^H signatures in the growing feathers (Singh et al., 2021). To estimate the latitude and photoperiod for each corresponding δ^2^H value, we obtained North America hydrogen isotope ratios for August month precipitation (δ^2^H_p_) from the OPIC 3.2 database (Bowen, 2020). For each bird, we found all feather δ^2^H values in the North America compatible to δ^2^H precipitation values (for detailed methods see Singh et al., 2021). The δ^2^H values were used as a continuous variable against all the physiological, hormone measures, and expressed transcripts.

### Testosterone ELISA

We measured circulating baseline testosterone levels in blood plasma by puncturing the wing vein and collecting blood into heparinized capillary tubes. The blood samples were stored at 4 ^°^C and plasma was extracted by centrifuging blood samples at 3000 rpm for 15 min at room temperature. The plasma was separated using a Hamilton syringe and stored at −20 °C until assayed to measure testosterone. The plasma samples were thawed on ice, and 20 μL of plasma from each individual was aliquoted in glass tubes. Hormone was extracted using diethyl-ether mixed by agitating and incubation at room temperature for 20 min. After incubation, all tubes were snap frozen and the supernatant was immediately transferred to a fresh tube. This procedure was repeated three times to extract the hormone, and finally the tube was dried using high pressure liquid nitrogen. Assay buffer (250 μL) was added to each tube, and 100 μL of extracted hormone was used in duplicate wells to run the testosterone assay. Plasma testosterone was measured using high sensitivity testosterone ELISA kit (Enzo ADI-900-176) as per manufacturer’s protocol. All samples were placed on one plate, testosterone ELISA with intra-assay coefficient variability of 0.6% ± 0.06 (mean ± SE) and sensitivity of 2.6 pg/mL.

### Measurement of gene expression

#### RNA extraction and sequencing

A total of 13 brains (6 migrant and 7 resident juncos) hypothalamii were punched and homogenized in RLT buffer containing 1% 2-Mercaptoethanol (BME) on ice. Total RNA was extracted with AllPrep DNA/ RNA Universal kit (Qiagen, Cat. # 80224) according to manufacturer protocol. RNA concentration and Integrity (RIN; Schroeder et al., 2006) were determined on Agilent 2200 TapeStation system. Total RNA was submitted to the Center for Genomics and Bioinformatics at Indiana University for subsequent RNA quality check, RNA library preparation and Illumina Nexseq 500 (43 nucleotides, paired end reads).

#### Quality filtering and mapping

The paired-end reads were de-multiplexed using bcl2fastq version v2.20.0.422 and about 28.5 million reads, with a raw read length of 43 nucleotides were assigned to each library. Raw reads were adapter trimmed and quality filtered using Trimmomatic version 0.33 (Bolger et al., 2014) requiring a minimum base quality score of 20 averaged across a sliding window of 3 bases. Reads shorter than 20 bases post trimming were discarded. Trimmed reads were mapped to the dark-eyed junco draft genome assembly sequence using the RNA-Seq read aligner STAR version 2.5.3a (Dobin et al., 2013).

#### Differential gene expression analysis

The transcriptome was annotated against functionally annotated regions defining the potentially transcribed elements on the dark eye junco draft genome assembly homologous to protein sequences from zebra finch (*Taeniopygia guttata, version 3.2.4*), a Passeriformes species phylogenetically closer to junco, were identified and annotated using BLASTX (Pruitt et al., 2009). Counts for read pairs aligning uniquely to each of the annotated transcribed elements were generated using feature Counts version 1.6.3 (Liao et al., 2014) and were used to identify the potentially significantly differentially expressed regions between migrant and resident birds at 5% FDR using the Bioconductor R package DESeq2 version 1.12.3 (Love et al., 2014).

#### Statistical Analysis

All analysis was done using R (version 3.2.0). Differences in mean hydrogen isotope ratio and T levels were calculated using a non-parametric Student’s t-test (Mann-Whitney U test) between subpopulation means. We used the Pearson correlation method to identify any significant correlation between hydrogen isotope ratios, testosterone levels, and normalized Differentially expressed gene expression values. For statistical significance, alpha was set at 0.05. The Benjamini–Hochberg procedure with a failed discovery rate of 5% was used to correct for multiple tests (Waite et al., 2006).

## Results

### Differences in seasonal phenotypes on the verge of spring

**FIGURE 2:**
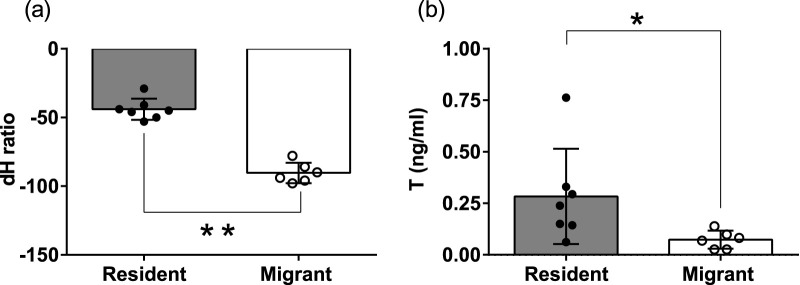
Scatter bar graph plot of feather hydrogen isotope (δ^2^H) value **(a)**, Plasma testosterone levels (ng/mL) **(b)**. Residents shown in the brown color bar, solid circles represent individual values. Migrants shown in white color bar, hollow circles represent individual values. An asterisk (*) on the line connecting the bars indicates a significant difference between groups (p < 0.05, Mann-Whitney U test). For statistical significance, alpha was set at 0.05.

Every year from fall to early spring, dark-eyed junco subspecies are sympatric and flock together at Mountain Lake Biological Station (MLBS), Virginia (Figures 1a,b). The pink-billed migrants arrive in the beginning of fall and depart to the north as spring approaches. Whereas blue-billed resident birds live year-round and breed at MLBS. In early spring, the migrants start preparing for migration. By the time resident juncos start showing gonadal recrudescence, the migrants are departing for their breeding ground. (Figure 1). The residents breeding and growing feathers at lower latitude had heavier δ^2^H than migrants (Mann-Whitney U test, p = 0.0012, Figure 2a). Also, there were significantly higher blood testosterone levels in resident as compared to migratory birds (Mann-Whitney U test, p = 0.014, Figure 2b). We did not find any significant difference in the body weight or subcutaneous fat scores of migrant and resident birds.

### Differentially expressed genes

**FIGURE 3:**
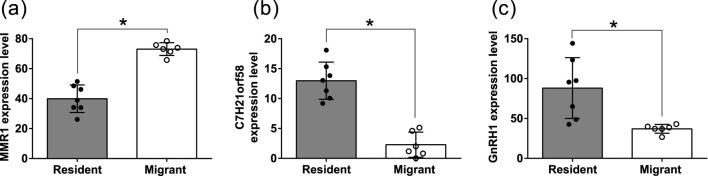
Transcriptome-wide differential gene expression (DGE). Box plot of significantly differentially expressed genes **(a–c)** in resident (solid circles) and migrant (hollow circles) hypothalamic transcriptome comparison. Each box plot represents the normalized expression value of transcripts. The false discovery rate multiple correction test was used to determine the significant change. For significance, the alpha was set at 0.05.

A total of 11,454 expressed sequenced tags (ESTs) were found after mapping to the dark-eyed junco draft genome assembly homologous to protein sequences from zebra finch (*Taeniopygia guttata*) as summarized in Supplementary Table S1. Further, we found three DEGs (FDR <0.05; Figure 3; Supplementary Table S1). Among the three DEGs, only one, gene macrophage mannose receptor 1-like (MMR1), was downregulated in resident birds (FDR = 0.0135; Figure 3a). Unlike MMR1, uncharacterized protein C21orf58 homolog (C7H21orf58; FDR = 0.0135; Figure 3b), gonadotropin releasing hormone 1 (GnRH1; FDR = 0.0216; Figure 3c) were upregulated in resident birds.

### DEGs correlation with δ^2^H isotope and plasma testosterone levels

**FIGURE 4:**
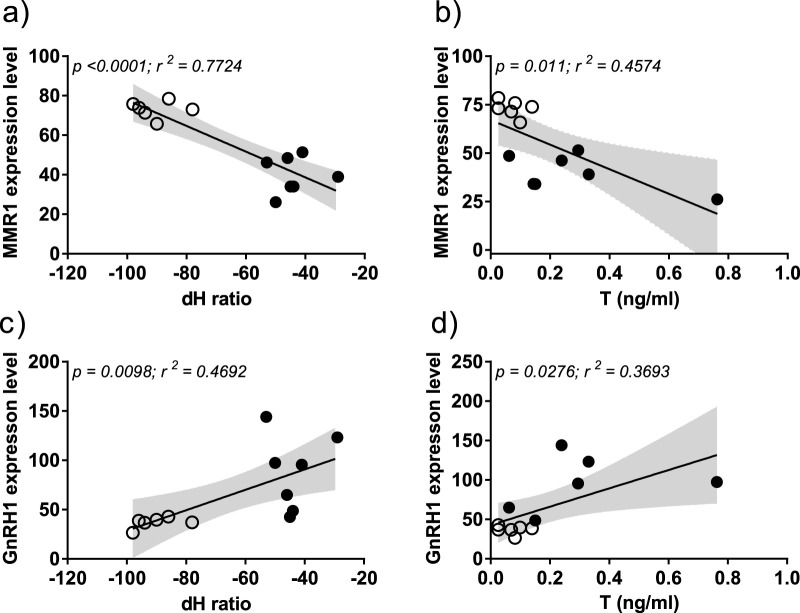
Correlation of DEGs with feather hydrogen isotope ratio (δ^2^H) and testosterone levels (T) in resident (solid circle) and migrant (hollow circles) juncos. X-axis represents the δ^2^H isotope values **(a–c)**, T levels in ng/mL **(b,d)**. Y-axis represents the normalized gene expression values **(a–d)**. Pearson’s correlation was used to determine the statistical significance; the alpha was set at 0.05. The positive and negative slope of regression line defines the direction of correlation between normalized gene expression levels, δ^2^H and T levels.

Macrophage mannose receptor 1 (MMR1) expression level showed a significant negative correlation to both δ^2^H isotope values (*r* ^2^ = 0.7724, p < 0.0001) and T levels (*r* ^2^ = 0.4574, p = 0.011; Figures 4a,b). In contrast, GnRH1 showed a positive correlation to both δ^2^H (*r* ^2^ = 0.4692, p = 0.0098) and T levels (*r* ^2^ = 0.3693, p = 0.0276; Figures 4c,d), indicating latitudinal differences in the HPG axis activation in anticipation to spring.

## Discussion

We compared the early spring transcriptome of neuroendocrine tissues (hypothalamus) derived from two populations of free-living dark-eyed juncos that reside in the same non-breeding location but differ in timing of reproduction and whether or not they migrate. We identified only three differentially expressed genes (GnRH 1, MMR1, C7H21orf58) in the resident and migrant junco hypothalamic tissue transcriptome in early spring. The most relevant gene to this study was GnRH1 encoding transcript, which was expressed at higher levels in early breeding residents than in migrant breeding at higher latitudes. A critical step in the hypothalamic-gonadal axis (HPG) induction is the release of GnRH1 that binds to gonadotrophic cells in the anterior pituitary to release luteinizing hormone (LH) and follicle-stimulating hormone (FSH) that goes in the circulation and binds to the gonads to release sex hormones (Cho et al., 1998). In addition to the differentially expressed genes (DEGs), we also found higher The hydrogen Prior research on this system using transcriptomics, proteomics, and quantification of specific target genes has shown variation in gene expression in tissues related to this phase lag in the timing of reproduction in migrants as compared to residents, including muscle, blood, and hypothalamus (Fudicker et al., 2016; Bauer et al., 2018; Singh et al., 2020). But to our knowledge, no other study has quantified gene expression in the hypothalamic-pituitary complex of two closely related sympatric populations of birds living in the wild during their divergence in reproductive timing and migratory preparedness under the same photoperiod and weather conditions in the wintering ground.

### Latitude of origin is related to the timing of gonad recrudescence

Populations of dark eyed junco are distributed across different latitudes in North America and vary widely in their phenotypic (body size, feather color, eye color) and migratory behavior as well as their reproductive phenologies (Nolan et al., 2002). Those breeding at higher latitudes are more likely to be migratory, and in spring, these higher latitude birds increase body fattening in preparation for migration. Juncos breeding at lower latitudes are more likely to be resident, and these non -migratory birds start to grow their gonads earlier in the year in response to the increase in early spring daylengths (Rogers et al., 1993; Fudickar et al., 2016). After autumn migration, high and low latitude populations typically overwinter together in sympatry.

Feather stable hydrogen isotope ratios have been used as a proxy for breeding latitude in movement ecology studies (Rubenstein et al., 2002; Hobson, 2003; Maggini et al., 2016). Because juncos molt their feathers late in the breeding season before they migrate, analysis of feather hydrogen isotope (δ^2^H) ratios in sympatric dark-eyed juncos during the non-breeding season can provide an estimate of their breeding latitudes (Fudickar et al., 2016; Singh et al., 2021; Wanamaker et al., 2020). Birds with lighter hydrogen isotope ratios likely breed at higher latitudes and those with heavier hydrogen isotope ratios likely breed at lower latitudes (Fudickar et al., 2016; Singh et al., 2021).

Several studies on junco populations have used feather hydrogen isotope ratios to show the latitudinal cline in spring preparedness of reproductive processes on the overwintering ground (Fudickar et al., 2016; Kimmitt et al., 2019; Wanamaker et al., 2020). These studies comparing overwintering resident and migrant junco populations from two separate locations, Virginia and Colorado, indicate that lower latitude juncos begin to grow their gonads earlier, thus on shorter days, than high latitude juncos (Fudickar et al., 2016; Wanamaker et al., 2020). This difference is likely related to the difference in the threshold photoperiod required to stimulate the neuroendocrine system regulating reproduction (Singh et al., 2021).

Temperate birds preparing to reproduce sense the change in day length and begin to rise in circulating testosterone, but activation of the hypothalamic-pituitary-gonad axis precedes that. Populations breeding at higher latitudes need relatively longer critical photoperiod than lower latitude birds to activate their HPG axis, grow gonads, and increase the circulating testosterone levels (Singh et al., 2021). Several field and captive studies in junco populations show co-variation between hydrogen isotope ratios and elevation of circulating testosterone levels in response to external GnRH 1 challenge, suggesting a latitudinal cline in reproductive timing (Fudickar et al., 2016; Greives et al., 2016; Wanamaker et al., 2020; Singh et al., 2021). In our study, we found higher variability in circulating baseline testosterone and GnRH-1 transcript in resident juncos, suggesting a plausible effect of non-photic cues, such as food availability and exposure to predators, regulating the pace of reproductive maturity among resident birds (Jacobs and Wingfield, 2000; Chmura et al., 2019).

### Latitude of origin is related to early spring hypothalamic GnRH 1 mRNA expression

In spring temperate-zone birds induce their gonads to grow in response to increasing day length. As noted, birds breeding at higher latitudes typically delay gonadal recrudescence until the days are longer because they migrate prior to reproducing (Dawson and Goldsmith, 1983; Silverin et al., 1993; Dawson, 2013; Greives et al., 2016). This additional spring migratory phenology in migratory populations breeding at higher latitudes delays the activation of the HPG axis and uses all energy in preparing for the migratory process.

The preoptic area (POA), a key regulatory nucleus located in the hypothalamus, has neuronal populations that synthesize and release a neuropeptide, gonadotropin-releasing hormone (GnRH1). Transition from short to long day lengths is known to induce GnRH1 release from the hypothalamus that in turn stimulates the release of luteinizing hormone (LH) from gonadotroph cells in the anterior pituitary (Dawson et al., 1985; Dawson and Goldsmith, 1997; Millar, 2005). Increased levels of LH coming to the systemic blood circulation further stimulate gonadal recrudescence and synthesis of testosterone, a gonadal steroid (Wingfield and Farner, 1993; Norris, 2007). Based on the studies on seasonal breeding songbirds, GnRH1 is the most critical top hierarchical molecule in stimulation of HPG axis with its elevation eventually leading to gonadal recrudescence.

In many avian species, levels of hypothalamic GnRH1 have been shown to be elevated significantly in the breeding season as compared to the non-breeding season (Dawson, 2013; Bauer et al., 2018). Significant seasonal changes in the abundance of hypothalamic GnRH1 transcript/peptide have been found in several songbird species, such as house sparrow (*Passer domesticus;* Hahn and Ball, 1995; Stevenson and MacDougall-Shackleton, 2005), American tree sparrow (*Spizella arborea*; Reinert and Wilson, 1996), house finches (*Carpodacus mexicanus*; Cho et al., 1998); dark-eyed juncos (*Junco hyemalis*; Deviche et al., 2006; Greives et al., 2016; Fudickar et al., 2016) and rufous-winged sparrows (*Aimophila carpolis*; Small et al., 2008).

A prior study comparing captive resident and migrant juncos in a common garden demonstrated differential response of the HPG axis to the same day lengths despite exposure to similar photoperiod, food, and temperature in captive condition. At a given early spring day length, resident juncos showed higher GnRH mRNA expression levels than migrant juncos, providing evidence for difference in the HPG axis activation in individuals originating from different latitudes (Bauer et al., 2018).

In our study, the hypothalamic transcriptome data collected from early spring free-living overwintering junco populations showed higher GnRH mRNA expression in resident compared to migrant juncos. This indicates stimulation of GnRH neurons leading to earlier recrudescence in low-latitude residents as compared to high-latitude migratory juncos. The higher GnRH1 transcript levels in resident junco than migrants could be involved in earlier activation of the hypothalamic gonadal axis in residents than in migrant juncos. Thus, differential GnRH1 synthesis in junco populations experiencing similar environmental conditions suggests difference in the photic sensitivity of the neuroendocrine machinery regulating the timing of reproduction in junco populations breeding at different latitudes.

In a similar study of the neuroendocrine tissue proteome of resident and migrant junco populations collected in early spring showed key proteins involved in GnRH 1 transcription, translation, and post-translational modification, stability, and synthesis (Singh et al., 2020). In addition, a proteome study on the juncos reported on here showed differential expression of Vasoactive Intestinal Peptide (VIP), a key neuropeptide molecule that has been implicated in the photoperiodic reproductive response and as a synchronizer of key circadian pacemaker cells in both birds and mammals (Singh et al., 2020; Rastogi et al., 2014).

Research on female phenology as compared to males is lacking. A study comparing female junco populations in Virginia showed similar trends of higher expression of key reproductive genes in the ovary and ovary mass in resident juncos than high-latitude migrants (Kimmitt et al., 2019). Future work should focus on investigating the role of GnRH1 and other key genes discussed above in the seasonal divergence of reproductive timing, comparing sex and population-level differences in both free-living and captive conditions.

In addition to GnRH 1, we found two more genes differentially expressed in the hypothalamus of resident and migrant juncos. Macrophage mannose receptor 1-like (MMR1), which encodes a glycan-binding protein expressed in macrophages, was downregulated in resident juncos. The MMR1 role has been studied in several biological processes that include the regulation of circulating reproductive hormone levels, homeostasis, innate immune response, and neuroinflammation (Cummings, 2022). The protein encoded from Mannose receptor gene is involved in the clearance of circulating Luteinizing hormone (LH) from the bloodstream which could lead to a decrease in the circulating testosterone levels (Cummings, 2022). Though in our study, we found a negative correlation of MMR1 transcripts level with testosterone levels. But given the low sample size and high variability in resident testosterone levels, we would avoid deriving any mechanistic relationship between MMR1 transcript levels regulating circulating testosterone levels. Another gene, C21orf58 homolog (C7H21orf58), which is an uncharacterized protein, showed higher transcript levels in resident birds. Not much is known about the function of the C7H21orf58 gene and its role in regulating the seasonal phenologies of birds.

## Conclusion

Based on transcriptomic differences in neuroendocrine tissue and circulating levels of testosterone, along with stable hydrogen isotope data revealing breeding latitude, we conclude that the HPG axis is activated earlier in the year in resident juncos that breed at lower latitudes as compared to migrant juncos that breed at higher latitudes. Future studies should focus on locating the neuronal cell bodies of GnRH 1 peptide and comparing its expression in winter non-breeding state and early spring divergent state when residents prepare to breed, while migrants prepare to migrate and delay breeding until they reach a higher latitude. In addition, future studies should extend to include other life-history states, the non-breeding wintering state, and the breeding phase at different latitudes.

The data presented in this study show the latitudinal effect on the timing of reproduction mediated via early activation of the HPG axis in birds that breed and develop at lower latitudes compared to higher latitude birds that breed in extreme long photoperiod. It will be interesting to explore the stimulation of the HPG axis at population-specific threshold photoperiod, where lower latitude resident juncos need relatively shorter photoperiod to induce gonads, and higher latitude migrants wait until they reach a critical photoperiod.

## Data Availability

The sequencing data are available in the NCBI Gene Expression Omnibus repository and are accessible through the GEO accession number # GSE305857.
